# Risk of diabetes after para-aortic radiation for testicular cancer

**DOI:** 10.1038/s41416-018-0248-x

**Published:** 2018-10-09

**Authors:** Harmke J. Groot, Jourik A. Gietema, Berthe M. P. Aleman, Luca Incrocci, Ronald de Wit, J. Alfred Witjes, Gerard Groenewegen, Peter de Brouwer, Otto W. M. Meijer, Maarten C. C. M. Hulshof, Hetty A. van den Berg, Tineke J. Smilde, Ben G. L. Vanneste, Maureen J. Aarts, Alphonsus C. M. van den Bergh, J. Martijn Kerst, Alexandra W. van den Belt-Dusebout, Sjoukje Lubberts, Katarzina Jóźwiak, Simon Horenblas, Flora E. van Leeuwen, Michael Schaapveld

**Affiliations:** 1 Department of Epidemiology, Netherlands Cancer Institute, Plesmanlaan 121, 1066 CX Amsterdam, The Netherlands; 2 Department of Medical Oncology, University Medical Center Groningen, Hanzeplein 1, 9713 GZ Groningen, The Netherlands; 3 Department of Radiation Oncology, Netherlands Cancer Institute, Plesmanlaan 121, 1066 CX Amsterdam, The Netherlands; 4 Department of Radiation Oncology, Erasmus Medical Center Cancer Institute, ‘s-Gravendijkwal 230, 3015 CE Rotterdam, The Netherlands; 5 Department of Medical Oncology, Erasmus Medical Center Cancer Institute, ‘s-Gravendijkwal 230, 3015 CE Rotterdam, The Netherlands; 6 Department of Urology, Radboud University Medical Center, Geert Grooteplein Zuid 10, 6525 GA Nijmegen, The Netherlands; 7 Department of Medical Oncology, University Medical Center Utrecht, Heidelberglaan 100, 3584 CX Utrecht, The Netherlands; 8 Department of Radiation Oncology, Dr. Bernard Verbeeten Institute, Brugstraat 10, 5042 SB Tilburg, The Netherlands; 9 Department of Radiation Oncology, VU University Medical Center Amsterdam, De Boelelaan 1117, 1081 HV Amsterdam, The Netherlands; 10 Department of Radiation Oncology, Amsterdam Medical Center, Meibergdreef 9, 1105 AZ Amsterdam, The Netherlands; 11 Department of Radiotherapy, Catharina Hospital, Michelangelolaan 2, 5623 EJ Eindhoven Eindhoven, The Netherlands; 12 Department of Medical Oncology, Jeroen Bosch Hospital, Den Bosch, Henri Dunantstraat 1, 5223 GZ ‘s-Hertogenbosch, The Netherlands; 13 Department of Radiotherapy, MAASTRO-clinic, Dr. Tanslaan 12, 6229 ET Maastricht, The Netherlands; 14 Department of Medical Oncology, Maastricht University Medical Centre, P. Debyelaan 25, 6229 HX Maastricht, The Netherlands; 15 Department of Radiation Oncology, University Medical Center Groningen, Hanzeplein 1, 9713 GZ Groningen, The Netherlands; 16 Department of Medical Oncology, Netherlands Cancer Institute, Plesmanlaan 121, 1066 CX Amsterdam, The Netherlands; 17 Department of Medical Oncology, University Medical Center Groningen, Hanzeplein 1, 9713 GZ Groningen, The Netherlands; 18 Department of Biostatistics, Netherlands Cancer Institute, Plesmanlaan 121, 1066 CX Amsterdam, The Netherlands; 19 Department of Urology, Netherlands Cancer Institute, Plesmanlaan 121, 1066 CX Amsterdam, The Netherlands

**Keywords:** Type 2 diabetes, Epidemiology, Testicular cancer

## Abstract

**Background:**

While the risk of diabetes is increased following radiation exposure to the pancreas among childhood cancer survivors, its association among testicular cancer (TC) survivors has not been investigated.

**Methods:**

Diabetes risk was studied in 2998 1-year TC survivors treated before 50 years of age with orchidectomy with/without radiotherapy between 1976 and 2007. Diabetes incidence was compared with general population rates. Treatment-specific risk of diabetes was assessed using a case–cohort design.

**Results:**

With a median follow-up of 13.4 years, 161 TC survivors were diagnosed with diabetes. Diabetes risk was not increased compared to general population rates (standardised incidence ratios (SIR): 0.9; 95% confidence interval (95% CI): 0.7–1.1). Adjusted for age, para-aortic radiotherapy was associated with a 1.66-fold (95% CI: 1.05–2.62) increased diabetes risk compared to no radiotherapy. The excess hazard increased with 0.31 with every 10 Gy increase in the prescribed radiation dose (95% CI: 0.11–0.51, *P* = 0.003, adjusted for age and BMI); restricted to irradiated patients the excess hazard increased with 0.33 (95% CI: −0.14 to 0.81, *P* = 0.169) with every 10 Gy increase in radiation dose.

**Conclusion:**

Compared to surgery only, para-aortic irradiation is associated with increased diabetes risk among TC survivors.

## Introduction

Several studies among childhood cancer and Hodgkin lymphoma survivors have reported that infradiaphragmatic radiotherapy may increase risk of diabetes.^1–4^ A recent study by van Nimwegen and colleagues showed that Hodgkin lymphoma survivors treated with para-aortic irradiation at doses of ≥36 Gray had a 1.8-fold increased risk of diabetes compared to patients without radiotherapy.^4^ Lower doses, which are more common in testicular cancer (TC) treatment, did not significantly increase diabetes risk in this study.

Although para-aortic radiotherapy (PAO-RT) is no longer used for treatment of non-seminoma patients nowadays and only rarely for treatment of stage I seminoma patients, it has long been part of TC treatment. PAO-RT for TC will generally result in irradiation of the head and body of the pancreas, which contains part of the insulin-producing beta-cells.^5^ Only one previous study assessed diabetes prevalence after radiotherapy for testicular cancer. Haugnes et al. observed a diabetes prevalence of 10.2% among patients treated with radiotherapy in 1980–1994 and screened for (risk factors for) cardiovascular disease (CVD) in 2008, which was 2.3-fold higher compared to prevalence among general population controls.^6^ The effect of radiation dose on diabetes risk was not assessed in this study. It is important to ascertain whether radiotherapy is associated with increased diabetes risk among TC survivors, since diabetes is an important predictor of premature mortality^7^ and may increase risk of coronary artery disease.^8,9^ Establishing a dose–response relationship is clinically important since radiation doses to organs at risk can be significantly reduced with modern radiation techniques and dose constraints to the pancreas can be introduced in radiation planning for TC and other malignancies. Therefore, this study aims to assess the association of radiation dose and diabetes risk after PAO-RT in a large multicenter cohort of TC survivors.

## Methods

### Study population and design

A hospital-based cohort was established including 6312 TC patients who were treated from January 1976 to December 2007 in 13 Dutch hospitals and were younger than 50 years at TC diagnosis. This cohort included 1874 5-year TC survivors treated between 1976 and 1995, entered in previous studies on cardiovascular disease (CVD) risk among TC survivors.^10,11^ Patients were identified through tumour registries and through the Netherlands Cancer Institute. Details on patient selection are presented in Supplemental Figure S1. For all patients in our hospital-based cohort, basic tumour and primary treatment characteristics were available.

In the current study, we aim to assess risk of diabetes among patients treated with radiotherapy as part of their primary treatment compared to risk of diabetes among patients treated with surgery only. Therefore, we first selected all patients from the hospital-based cohort who received either surgery only or surgery with radiotherapy as part of primary treatment and were aged between 12 and 50 years at orchidectomy and were alive 1 year after TC diagnosis (*N* = 3746, Supplemental figure S1). For 68.6% of the cohort (*N* = 2568) and for 76.7% of the subcohort (*N* = 544), diabetes and cardiovascular disease follow-up was available through the GP and/or medical files until at least January 2011. We excluded all patients without any cardiovascular disease follow-up (575 patients); those who died or were lost to follow-up within 1 year (71 patients), those who received chemotherapy in the first year after TC diagnosis (85 patients) and who developed diabetes before TC (1 patient) or within 1 year after TC diagnosis (16 patients). This left 2998 patients for analysis. We subsequently identified all patients who had developed diabetes in our study cohort (*N* = 161, Supplemental figure S1).

Similarly, we selected all patients from the subcohort who had received either surgery only or surgery with radiotherapy as primary treatment and who had survived at least 1 year after TC diagnosis (*N* = 614).

We used a case-cohort design to facilitate detailed treatment data collection while allowing assessment of multiple treatment-associated outcomes.^12^ A hospital-stratified subcohort comprising 15% of the base cohort (25% in the two coordinating hospitals) was randomly selected, without taking cardiovascular or oncological follow-up into account. This subcohort comprised 1175 TC patients. For all randomly sampled patients in the subcohort and for all patients who developed diabetes (which includes patients who developed diabetes and were sampled in the subcohort), detailed treatment data were abstracted from the medical charts, including chemotherapy regimens, number of cycles and cumulative doses, radiotherapy fields and doses for primary treatment as well as relapse treatment. The study protocol was submitted to the Institutional Review Board of the Netherlands Cancer Institute, which waived the requirement for individual patient consent.

### Treatment

All patients underwent orchidectomy. Radiotherapy for stage I and II seminomas typically comprised irradiation of the infradiaphragmatic para-aortic, ipsilateral, iliacal and inguinal lymph nodes. Irradiation of the para-aortic and iliac lymph nodes constitutes the dogleg radiation field.^11,13^ For left-sided tumours the para-aortic fields were usually extended to include the left renal hilar nodes. The radiation dose used in the treatment of seminoma patients gradually declined over time from 30 to 35 Gray in the 1980s to 26 Gray from 1990 onwards.^10,14^ Since the mid-2000s, stage I seminoma patients are increasingly treated with either surveillance, or in case of at least two risk factors, 20 Gray radiotherapy or 1 cycle of carboplatin (AUC 7).^15–17^ Before 1985, treatment for stage I or IIA non-seminoma frequently included adjuvant radiotherapy with a dose of 40–50 Gray in 20–25 fractions.^13,14^ From 1985 onwards, stage I non-seminoma patients were generally treated under watchful waiting protocols, depending on disease severity and prognostic factors.^13,17^

### Outcome assessment

Information on date of diagnosis, type of and treatment for diabetes, was obtained between 2013 and 2016 from the medical records and/or questionnaires sent to the patient’s general practitioner (GP) and from medical correspondence with the treating physician. In Dutch guidelines for general practitioners, diabetes is typically defined as fasting plasma glucose ≥7.0 mmol/l.^18^ Population screening for diabetes is not part of current Dutch GP guidelines, hence, diabetes case ascertainment was based on clinical presentation to regular care (see Supplement S2).

### Statistical analysis

Time at risk started 1 year after start of TC treatment and ended at date of diabetes diagnosis, date of death or date of last follow-up, whichever occurred first. Patients who relapsed were censored at the date of relapse if treatment for the relapse included chemotherapy and relapse treatment was started 1 year or more after TC diagnosis. The last medical follow-up as recorded in the medical files was used when no follow-up information from GP questionnaires was available. In the cohort, follow-up for diabetes and other cardiovascular disease risk factors was derived from information from the medical record and the GP in 32.2%, from the GP only in 61.4% and from the medical record only in 6.4% of the patients; in the subcohort these percentages were 81.6%, 2.9% and 15.5%, respectively. In total, 11.2% of the patients with diabetes in the subcohort (four patients) were identified through information from the medical record only.

#### Comparison with the general population

Incidence of diabetes was compared with sex-, age- (5-year strata), and calendar year-specific diabetes incidence rates in the Dutch population. Diabetes incidence rates for the Dutch population were obtained from electronic health records of general practitioners participating in NIVEL Primary Care Database and were available for 2002–2016 (Number NZR-00316.030). Therefore, analyses were left censored at January 1, 2002. For more details on diabetes population, reference rates and calculation of standardised incidence ratios we refer to Supplemental file S3.

#### Case-cohort comparisons: assessing the relationship between treatment and diabetes risk

Cumulative incidence of diabetes was estimated in the presence of death as competing risk, and trends over time were evaluated using competing risk regression models with adjustment for age.^19^ Missing information on covariates were imputed using chained equations (MICE), ignoring patient clusters, and creating 20 datasets^18,19^. Disease stage, radiation field and smoking were imputed with multinomial logistic regression, while weight, height and radiation dose were imputed using linear regression. In all imputation models, year and age at treatment, histology, age, hospital of diagnosis, diabetes status and cumulative hazards of diabetes calculated with a Cox regression model before imputation, were included as extra covariates. Associations of TC treatment with diabetes risk were assessed using multivariable Cox regression models with time since TC treatment as time scale. Barlow’s inverse probability weights were used to adjust the partial likelihood function for our case-cohort design^12^. TC treatment was entered in the model as a time-varying variable. Effects of treatment were evaluated accounting for the effects of other covariates where appropriate. Modification of a radiation-associated diabetes risk was assessed by inclusion of interaction terms for age, obesity (BMI > 30 kg/m^2^) and smoking with radiotherapy. To assess an excess relative risk for prescribed radiation dose in relation to diabetes risk in our case-cohort setting, the linear increase in the HRs over categories of prescribed dose was estimated separately for each imputed data set using weighted least square regression with weights equal to 1/variance of each HR and subsequently averaged over all 20 data sets. Cox regression model estimates from the imputed data sets were pooled using Rubin’s rule.^20^ Dose categories were chosen such that each category represented at least five diabetes cases (0, ≤24, 25, 26, 27–30, 31–35, 36–40 and ≥40 Gray) and confidence intervals of the HRs were based on Wald test. The proportional hazards assumption was assessed using residual-based methods. STATA (version 13.1, Statacorp LP, College Station, TX) statistical software was used for analysis. A *P* ≤ 0.05 was considered significant.

## Results

### Patient characteristics

Our study cohort comprised 2071 (69.1%) seminoma and 927 non-seminoma TC patients (30.9%). Median age at TC treatment was 33.3 years (Interquartile range (IQR): 27.9–39.0 years). The median follow-up was 13.4 years, during which 161 TC patients (5.3%) developed diabetes. For 29.6% of the TC patients, follow-up exceeded 20 years. Among patients with diabetes, the median interval until diabetes was 14.3 years (IQR: 8.9–21.1 years). The median age at TC treatment was 37.4 years (IQR: 32.1–42.0 years) for patients with diabetes; the median age at diabetes diagnosis was 51.9 years (IQR: 45.1–59.2 years).

Of all patients in the subcohort, comprising 414 seminoma (67.4%) and 200 non-seminoma patients (32.6%), 406 patients (66.1%) had received radiotherapy (Table 1). Median follow-up was 15.8 years (IQR: 8.4–23.4 years) for patients treated with surgery only and 11.8 years (IQR: 6.2–19.8 years) for patients treated with radiotherapy. Of the seminoma patients in the subcohort, 6.3% underwent only surgery. The median radiotherapy dose to the para-aortic field was 30 Gray (range: 16–70 Gray) in 1976–1985 and decreased to 26 Gray (range: 17–51 Gray) during 1986–1995 (Supplemental table S4 and supplemental figure S5). Of the non-seminoma patients treated before 1985, 31.9% received infradiaphragmatic radiotherapy with a median dose of 40 Gray (range: 30–70).

**Table 1:** Patient characteristics for 1-year testicular germ cell cancer survivors treated between 1976 and 2007 with primary surgery with/without radiotherapy

Patient characteristic	Base cohort		Subcohort^a^		Diabetes after TC	
	*N*	%	*N*	%	*N*	%
All patients	2998		614		161	
Histology						
Seminoma	2071	69.1	414	67.4	131	81.4
Non-seminoma	927	30.9	200	32.6	30	18.6
Age at testicular cancer, years						
Median±IQR	33.3	27.9–39.0	33.3	28.0–39.0	37.4	32.1–42.0
<30	1012	33.8	213	34.7	28	17.4
30–40	1349	45.0	271	44.1	73	45.3
40–50	637	21.3	130	21.2	60	37.3
Treatment period						
1976–1985	477	15.9	93	15.2	63	39.1
1986–1995	916	30.6	216	35.2	50	31.1
1995–2007	1605	53.5	305	49.7	48	29.8
Follow-up duration, years						
Median±IQR	13.4	8.3–20.5	14.6	9.6–21.3	14.3	8.9–21.1
0–4 years	153	5.2	43	7.0	18	11.2
5–9 years	641	21.4	126	20.5	36	22.4
10–14 years	738	24.6	149	24.3	34	21.1
15–19 years	578	19.3	107	17.4	23	14.3
20–24 years	434	14.5	101	16.5	24	14.9
25–29 years	243	8.1	52	8.5	16	9.9
≥30 years	211	7.0	36	5.9	10	6.2
Relapse, % yes	79	2.6	30	4.9	3	1.9
Treatment for TC (total treatment)						
Hemi-orchidectomy only	—	—	208	33.9	28	17.4
Radiotherapy	—	—	406	66.1	133	82.6
RT field						
No PAO/ DL radiotherapy^b^	—	—	208	33.9	28	17.4
Radiotherapy, PAO	—	—	164	26.7	39	24.2
Radiotherapy, DL	—	—	229	37.3	87	54.0
Unknown	—	—	13	2.1	7	3.7
Prescribed PAO-radiation dose						
No PAO radiotherapy ^b^	—	—	208	33.9	28	17.4
<26 Gray	—	—	86	14.0	34	21.1
26–32 Gray	—	—	233	38.0	69	42.9
≥32 Gray	—	—	48	7.8	17	10.6
Dose unknown	—	—	26	4.2	6	3.7
Field unknown	—	—	13	2.1	7	4.3
Median dose (IQR)			26	26-26	26	25-30
Cardiovascular disease risk factors						
BMI at TC diagnosis						
<30 kg/m^2^	—	—	215	35.0	57	34.4
≥30 kg/m^2^	—	—	21	3.4	15	9.3
Unknown	—	—	378	61.6	89	55.3
Diabetes, % yes^c^			39	6.4	161	100
Diabetes type						
Insulin dependent	—	—	3	7.7	24	14.9
Non-Insulin dependent	—	—	32	82.0	123	76.4
Unknown type or treatment	—	—	4	10.3	14	8.7
Hypercholesterolemia at TC diagnosis						
Yes	—	—	5	0.8	3	1.9
No	—	—	574	93.5	141	87.6
Unknown	—	—	35	5.7	17	10.6
Hypertension at TC diagnosis						
Yes	—	—	10	1.6	9	5.6
No	—	—	568	92.5	136	84.5
Unknown	—	—	36	5.9	16	9.9
Smoking status at TC diagnosis						
Yes	—	—	181	29.5	55	34.2
No	—	—	229	37.3	74	46.0
Unknown	—	—	204	33.2	32	19.9
Vital status at date of last contact						
Alive	2884	95.6	587	95.6	135	83.9
Died	97	3.2	22	3.6	26	16.2
Emigrated	37	1.2	5	0.8	0	0
Age at end of follow-up						
Median±IQR	48.2	40.9–55.7	47.4	39.7–54.7	51.9	45.1–59.2

*PAO* para-aortic field, *DL* Dog-Leg field, *BMI* body mass index (in kg/m^2^)

^a^In the subcohort 39 patients had developed diabetes (6.4% of subcohort)

^b^includes infradiaphragmatic fields other than para-aortic radiotherapy only or dog leg, i.e. iliac/inguinal or scrotal radiation fields.

^c^7 of the 161 diabetes cases developed a pancreatic carcinoma before (*N*=2) around (*N*=4) or after (*N*=1) their diabetes diagnosis. Median time to diabetes was 17.8 years (IQR: 11.1–26.1) for these patients

### Diabetes risk compared to the general population: case-cohort analysis

Diabetes risk among TC patients did not differ from the diabetes risk expected based on incidence rates for the general Dutch male population (standardised incidence rate (SIR): 0.9, 95% CI: 0.7–1.1, Supplemental table S6). The SIR for diabetes was not increased for patients treated with radiotherapy (SIR: 1.0, 95% CI: 0.8–1.2) and diabetes risk did not differ by age at TC diagnosis (P-heterogeneity: 0.970). SIRs were similar in the full cohort analysis, although risk was lower than expected for non-irradiated patients (SIR: 0.4, 95% CI: 0.3–0.6).

### Diabetes risk after infradiaphragmatic radiotherapy: case-cohort analyses

**Fig. 1:**
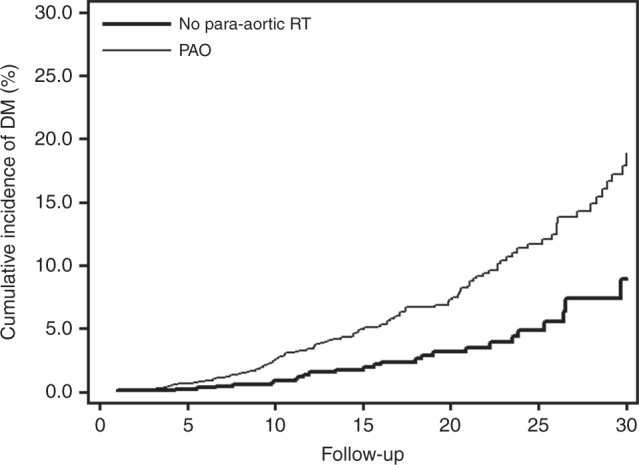
Incidence of diabetes among testicular cancer survivors treated with para-aortic radiotherapy compared to orchidectomy only. PAO para-aortic irradiation. Diabetes incidence was significantly increased after para-aortic radiotherapy (*P* < 0.001)

The cumulative incidence of diabetes was 6.1% (95% CI: 5.0–7.4%) at 20 years and 15.6% (95% CI: 12.4–19.2%) at 30 years after TC treatment. Adjusted for age at TC diagnosis patients treated with para-aortic radiotherapy had a significantly higher risk of diabetes than patients who underwent only surgery (*P* < 0.001; Fig. 1). Patients with a prescribed radiation dose of >26 Gray to a para-aortic field had a 30-year cumulative incidence of 16.7% (95% CI: 11.9–22.2%), compared to a 30-year cumulative incidence of 9.5% (95% CI: 5.0–15.7%) for patients treated with surgery only (*P* value: 0.122, adjusted for age).

**Fig. 2:**
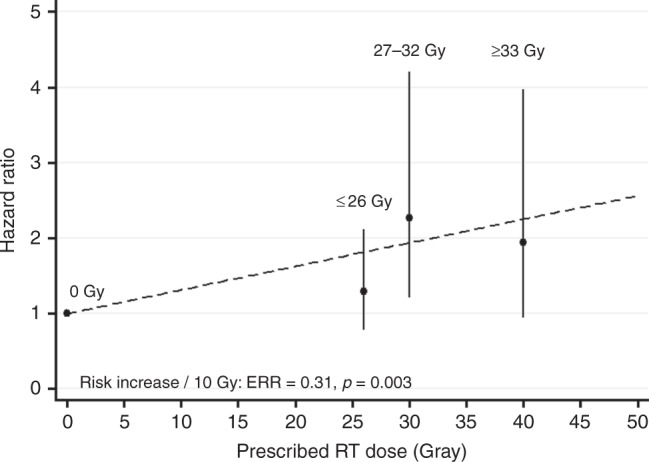
Risk of diabetes by prescribed para-aortic radiation dose among testicular cancer survivors. HRs for diabetes risk for prescribed dose categories are plotted at the mean dose within each category (26 Gy for ≤26GY, 30 Gy for 27–32 Gy and 40 Gy for ≥33 Gy). Vertical lines represent the 95% CI for each category of dose. The HRs for determining the excess risk increase per 10 Gray (ERR) were derived from a model with adjustment for age and BMI continuously. The excess risk was estimated based on the following dose categories: 10–24 Gy, 25 Gy, 26 Gy, 27–30 Gy, 31–39 Gy and ≥40 Gy, with median doses of 21, 25, 26, 30, 33, 40 and 50 Gy

Adjusted for age, para-aortic RT was associated with a 1.66-fold (95% CI: 1.05–2.62; Table 2) increased diabetes risk compared to no radiotherapy. When adjusting for BMI and age (as continuous variables), risk decreased to 1.54 (95% CI: 0.96–2.45). A radiation dose of ≤26 Gray to the para-aortic field was associated with a 1.29-fold (95% CI: 0.79–2.11) increased diabetes risk while doses of 27–32 Gray and ≥33 Gray were associated with a 2.26-fold (95% CI: 1.22–4.21) and a 1.94-fold (95% CI: 0.95–3.97) increased diabetes risk, respectively (P-trend: 0.011). The excess hazard increased with 0.31 with every 10 Gray increase in the prescribed radiation dose (95% CI: 0.11–0.51, *P* = 0.003, Fig. 2). The excess risk among irradiated patients increased with 0.33 (95% CI: −0.14 to 0.81, *P* = 0.169) with every 10 Gy increase in radiation dose.

**Table 2:** Multivariable analysis for diabetes risk by treatment among TC survivors (case-cohort study with detailed treatment data)

	Adjusted for age			Adjusted for age and BMI at TC diagnosis (continuously)	
	n/*N*	HR	95% CI	HR	95% CI
Radiotherapy^a^					
No	27/196	1	ref	1	ref
Yes	134/379	1.66	1.05–2.62	1.54	0.96–2.45
* P*-_heterogeneity_			0.030		0.074
Radiotherapy field ^a^					
No PAO-radiotherapy	27/196	1	ref	1	ref
PAO-radiotherapy, left(incl. bilateral)	58/172	1.62	0.99–2.68	1.46	0.87–2.46
PAO-radiotherapy, right ^b^	74/199	1.71	1.05–2.77	1.61	0.98–2.64
Other infradiaphragmatic fields	2/8	1.26	0.25–6.45	1.16	0.24–5.67
* P* -_heterogeneity_			0.156		0.204
Radiotherapy dose^a^					
No para-aortic radiotherapy	27/196	1	ref	1	ref
≤26 Gray	80/281	1.45	0.91–2.32	1.29	0.79–2.11
27–32 Gray	35/46	2.39	1.31–4.35	2.26	1.22–4.21
≥33 Gray	19/52	1.88^c^	0.92–3.86	1.94^c^	0.95–3.97
* P* -_heterogeneity_			0.024		0.028
* P*_-trend_			0.013		0.011
BMI at TC diagnosis					
<30 kg/m^2^	129/531	1	ref	—	—
≥30 kg/m^2^	32/44	2.52	1.31–4.87	—	—
* P* -_heterogeneity_			*P*<0.001		
Smoking status at TC diagnosis					
No	94/330	1	ref	—	—
Yes	67/245	0.94	0.64-1.37	—	—
* P* -_heterogeneity_			0.625		

n/*N* Median number of cases/median number of non-cases in the 20 imputed and pooled data sets

*PAO* para-aortic radiotherapy, *BMI* body mass index (kg/m^2^)

^a^Primary and/or follow-up treatment

^b^Diabetes risk was not significantly different between left and right sided PAO irradiation, *P*-heterogeneity, adjusted for age: 0.084; P-heterogeneity, adjusted for age and BMI: 0.489

^c^Para-aortic RT dose 27-32 Gy and >32 Gy versus ≤26 Gy, *P*-hetero ≤0.016 and *P*-hetero ≤0.093, respectively (median dose: 26 Gy in category ≤26Gy, 30 Gy in category 27–32 Gy and 40 Gy in category ≥33 Gy)

Obesity at TC diagnosis was an independent risk factor for diabetes, associated with a 2.52-fold (95% CI: 1.31–4.87) increased diabetes risk, but did not modify the association between radiotherapy and diabetes risk (*P*-interaction = 0.496). The effect of infradiaphragmatic radiotherapy on diabetes risk was also not modified by smoking or age. Complete case analyses, which only included patients who had no missing data on radiation fields and dose, provided similar results (Supplemental table S7).

## Discussion

In this large cohort study we observed a 1.66-fold increased risk of diabetes among TC survivors treated with para-aortic radiotherapy compared to TC survivors treated with surgery only, adjusted for age. Diabetes risk increased with a higher para-aortic radiation dose. However, diabetes risk after exposure to radiation was not increased compared to that of males in the Dutch general population, while diabetes risk was lower than expected among survivors treated with surgery only.

Four studies previously investigated diabetes risk after para-aortic radiotherapy in cancer survivors.^1,2,4^ In a French–British cohort, de Vathaire and colleagues found an 11-fold increased diabetes risk among childhood cancer survivors irradiated with 20 Gray to the pancreatic tail compared to those not treated with radiotherapy; risk increased with increasing dose up to ≥30 Gray. Meacham and colleagues observed a 1.8-fold increased risk of developing diabetes among childhood cancer survivors compared to sibling controls.^2^ In particular, high diabetes risks were observed after treatment with radiotherapy, total body irradiation, alkylating agents and treatment at ages <4 years. Among HL survivors, van Nimwegen and colleagues observed a 1.8-fold increased risk of diabetes after para-aortic radiotherapy with a prescribed dose of ≥36 Gray compared to no para-aortic radiotherapy.^4^ So far, only one cohort study investigated diabetes risk after radiotherapy for testicular cancer. Haugnes and colleagues found a higher prevalence of diabetes after radiotherapy exposure (10.2%, 38 diabetes patients) compared to surgery only (4.0%, 8 diabetes patients), but did not provide relative risk estimates.^6^ Although para-aortic radiotherapy for TC does not include the pancreatic tail and generally lower para-aortic doses are used compared to treatment of childhood cancer and HL patients, infradiaphragmatic radiotherapy was associated with an elevated diabetes risk in TC survivors. Somewhat surprisingly, patients treated with a para-aortic dose ≥33 Gray did not have a higher diabetes risk than patients who received doses ≤26 Gray. However, since only a small number of patients had doses ≥33 Gray, our power to reliably establish an increased diabetes risk within this dose category was low.

Diabetes induced by infradiaphragmatic radiotherapy may result from direct damage to the pancreatic beta-cell Islets of Langerhans^1,2,4^ or to the pancreatic microvasculature.^21,22^ A study in primates showed that pancreatic radiotherapy induced degranulation, vacuolisation, mitochondrial destruction and impaired insulin secretion shortly after treatment.^23^ Adjuvant radiotherapy has also been shown to reduce beta-cell function and insulin secretion capacity of the pancreas in 1-year gastric cancer survivors.^22^ Meacham and colleagues hypothesised that the radiotherapy effect is independent of obesity and therefore a result of beta-cell deficiency.^2^ Others found a marginally but not significantly larger diabetes risk among those with increased BMI.^1,4^ In our study, obesity at baseline was an independent risk factor for diabetes but did not modify radiotherapy-associated diabetes risk.

The pathway from para-aortic radiotherapy to diabetes could also be modified by subclinical hypogonadism after orchidectomy, which has been shown to increase the risk of developing a metabolic syndrome.^24^ Hypogonadism is associated with increased insulin insensitivity and diabetes risk.^25,26^ In the study of Haugnes and colleagues, a 2.3-fold increased odds for diabetes was observed after radiation compared to surgery only.^6^ Serum testosterone levels were lower after radiation compared to surgery only, but patients with orchidectomy only did not experience increased diabetes risk compared to general population controls.^6^ In our study the prevalence of hypogonadism was similar after radiation versus surgery (16% after orchidectomy only and 18% after radiotherapy). Therefore, this pathway does not appear to play a major role in diabetes etiology.

We observed a fairly large number of incident diabetes cases (*N* = 161). Our risk estimates may underestimate the true risk, as we may have missed asymptomatic diabetes cases. As a booster dose was not recorded, we may also have underestimated the true dose to the pancreas in some patients, which could have attenuated our risk estimates for the association with prescribed radiation dose. Our study provides reliable risk estimates for the association of PAO-RT and diabetes risk as we had access to detailed treatment data, including relapse treatment.

Survivors of testicular cancer could be more health-conscious than their peers in the general population which can have influenced their lifestyle following testicular cancer treatment. Since diabetes in part depends on life-style, this may explain the unincreased risk of diabetes among irradiated patients compared to the general population. We did not find evidence for a more healthy lifestyle at diagnosis among irradiated and non-irradiated patients in the subcohort. Prevalence of smoking did not differ between these patients and smoking at diagnosis was not associated with diabetes risk. Although BMI above 30 kg/m^2^ was associated with diabetes risk, prevalence of diabetes was similar in both groups. We found no evidence for confounding nor for modification of the radiation associated diabetes risk by BMI, although imputation was necessary since for approximately 40% of the patients either weight or height was missing. A limitation of our general population comparisons is left-censoring in the analysis due to lack of reference data before 2002, resulting in exclusion of many person-years from analysis, mainly affecting patients treated in the distant past who were more frequently irradiated.

Although infradiaphragmatic radiotherapy is currently largely abandoned in treatment of stage I seminoma, while stage II seminoma patients with increased risk of metastatic disease still have an indication for radiotherapy, the association of previous para-aortic irradiation with diabetes risk is still very relevant for many long-term TC survivors. Radiotherapy techniques have improved over the past decades, with increasing use of CT planning since the late ‘90s, which has resulted in more tailored abdominal RT fields. However, this did not necessarily decrease radiation field width in our study. In some cases, the amount of irradiated tissue near the renal hilum may even have increased over time. Currently, there are still no recommended constraints for the radiation dose to the pancreatic gland. We expect therefore that the change in the pancreatic volume included within the para-aortic radiation field over time was limited. In more recently irradiated patients diabetes risk may be lower due to the use of more precise intensity modulated radiotherapy techniques, exposing a lower proportion of the pancreas to a relatively high radiation dose. Nonetheless, this study suggests another reason why stage I seminoma patients may benefit from surveillance above undergoing PAO-RT, beyond the observation that the 5-year relapse rate of 4–6% for patients without prognostic risk factors under surveillance protocols^27,28^ is not that different from the 4% 5-year relapse rate after abdominal RT.^27,29^

In conclusion, TC survivors treated with para-aortic irradiation have a 1.66-fold higher risk of diabetes compared to TC survivors treated with surgery only. Although diabetes risk is only moderately increased after para-aortic irradiation, and the relative risk in our study is lower compared to the risks observed in childhood cancer survivors, early detection and treatment of diabetes may prevent or alleviate late vascular damage and CVD. With the current improvements in radiation techniques the dose to organs at risk can be reduced, especially if the pancreas is defined as an organ at risk which is also important in radiation treatment of other malignancies. Unfortunately the optimal dose constraint cannot be derived from this study, although our study suggests to keep the dose to the pancreas below 26 Gray. This novel finding does not only apply to irradiated TC patients, but potentially also to patients with abdominal lymphoma, cervical cancer or stomach cancer who receive abdominal irradiation.

## Electronic supplementary material

Supplements

## Data Availability

data were collected at NKI-AVL and are not publicly available.
