# Supplementary material for: Risk of diabetes after para-aortic radiation for testicular cancer
Source: Br J Cancer. 2018 Oct 9;119(7):901–7. doi: 10.1038/s41416-018-0248-x (PMC6189211; doi:10.1038/s41416-018-0248-x)
Supplement: Supplementary file 1 — Supplements [file 41416_2018_248_MOESM1_ESM.docx]

**Supplemental figures S1. Patient inclusion**

***Excluded for current study:***

458 patients, treated with CT as part of primary treatment (including 33 patients treated with radiotherapy & chemotherapy

8 patients, not treated for TC in one of the participating centers (secondary referrals for SMN treatment)

***Excluded for current study:***

2,566 patients, treated with CT as part of primary treatment (including 127 patients treated with radiotherapy & chemotherapy and including 19 patients not treated for TC in one of the participating centers (secondary referrals for SMN treatment)

**Cohort**

TC patients, aged 12-50 years at TC diagnosis,treated 1976-2007

N=6,312

1976-2007

N=

N=

**Randomly sampled subcohort**

15% for all hospitals, except two coordinating centers (25%) *N*=1,175

No primary chemotherapy

*N*=3,746

No primary chemotherapy

*N*=709

***Additionally excluded:***

19 patients without cardiovascular follow-up

57 patients treated with relapse chemotherapy within 1 year of TC diagnosis

3 DM cases diagnosed within 1 year of TC diagnosis

16 patients were lost to follow-upwithin 1 year

***Additionally excluded:***

575 patients without cardiovascular follow-up

71 patients lost to follow-up within 1 year

85 patients treated with relapse chemotherapy within 1 year of TC diagnosis

1 DM case diagnosed before TC

16 DM cases diagnosed within 1 year of TC diagnosis

**Included in study**

1-year survivors

*N*=2,998

**Included in study**

1-year survivors

*N*=614

Diabetes incidence: N=39 (6.4%)

Diagnosed with Diabetes during follow-up

*N*=161 (5.4%)

*N*=122 (5.1%) in the cohort excluding subcohort patients

**Supplemental text S2. Estimation of Standardized incidence ratios**

The reference population increased from 279,561 patients represented by the practices of 69 general practitioners in 2009 to 1,660,702 patients represented by 416 general practitioners practices in 2015. A loglinear regression model was used to smooth the calendar-year and age-specific (5-year age strata) DM incidence rates for the Dutch male population from 2002 onwards. Incidence of DM in our case-cohort study was compared with smoothed sex-, age- (5-year strata), and calendar year–specific DM incidence rates in the Dutch population. Similarly incidence rates for DM in the full cohort were also compared with sex-, age-, and calendar year-specific DM incidence rates in the Dutch population. Standardized incidence ratios (SIRs) were calculated as the ratios of the observed to expected number of patients with DM in our study, the latter weighted with the inverse subcohort sampling fraction. Tests for homogeneity and trend of SIRs by age, follow-up interval, attained age and treatment were performed within collapsed person-time Poisson regression models. Tests for trend in SIRs were performed by evaluating the likelihood of a model with a variable representing follow-up interval or attained age as discrete values against the likelihood of a model without that variable.

**Supplemental text S3. Dutch guidelines for cardiovascular risk factor screening in the general practice**

The general practitioner (GP) determines (preferably fasting) blood glucose levels in patients with symptoms or underlying diseases which are associated with diabetes mellitus, like excessive thirst, polyuria, weight loss, pruritus vulvae at older ages, recurrent urine tract infections and balanitis, mononeuropathy, neurologic pains and sensibility disorders.

In addition, patients older than 45 years who have one or more of the characteristics mentioned below, are advised to undergo a three-yearly GP visit for evaluation of blood glucose levels:

having a BMI ≥ 27 kg/m2;

diabetes mellitus type 2 was diagnosed among parents or siblings;

hypertension (systolic blood pressure > 140 mmHg of treatment for hypertension);

fatty acid metabolism disorders (HDL-cholesterol ≤ 0,90 mmol/l, triglycerids> 2,8 mmol/l);

increased risk of cardiovascular diseases (see guideline for cardiovascular risk management, in Dutch: NHG-Standaard Cardiovasculair risicomanagement);

Turkish, Moroccan or Surinam origin;

In addition to what was mentioned above, for patients with Hindustan origin a lower age limit of 35 years is used

| Supplemental tables S4a/4b. Treatment distribution by period in the DM subcohort. A: seminoma patients in subcohortfor DM study. B: All patients in subcohort for DM study. | | | | | | | | |
| --- | --- | --- | --- | --- | --- | --- | --- | --- |
| Panel A. Treatment by calendar period for seminomasin the subcohort | | | | | | | | |
|  | **Overall**  ***N*=414** | | **1976-1985**  ***N*=46** | | **1986-1995**  ***N*=143** | | **1996-2007**  ***N*=225** | |
|  | ***N*** | **%** | ***N*** | **%** | ***N*** | **%** | ***N*** | **%** |
| Median age at diagnosis (SD) | 35.5 | 30.7-40.2 | 31.0 | 26.8-41.9 | 35.4 | 29.8-40.9 | 35.2 | 31.1-39.9 |
| Follow-up duration, years |  |  |  |  |  |  |  |  |
| Median | 14.2 |  | 28.6 |  | 20.3 |  | 11.3 |  |
| IQR | 9.8-20.2 |  | 19.9-32.4 |  | 17.6-23.4 |  | 8.5-13.7 |  |
| 0-4 | 29 | 7.0 | 4 | 8.7 | 19 | 8.4 | 19 | 8.8 |
| 5-9 | 83 | 20.1 | 2 | 4.4 | 73 | 32.4 | 73 | 32.4 |
| 10-14 | 113 | 27.3 | 2 | 4.4 | 102 | 45.3 | 102 | 45.3 |
| 15-19 | 75 | 18.1 | 4 | 8.7 | 31 | 13.8 | 31 | 13.8 |
| 20-24 | 71 | 17.2 | 9 | 19.6 | - | - | - | - |
| 25-29 | 24 | 5.8 | 6 | 13.1 | - | - | - | - |
| 30+ | 19 | 4.6 | 19 | 41.3 | - | - | - | - |
| Relapse, % yes | 19 | 4.6 | 3 | 6.5 | 6 | 4.2 | 10 | 4.4 |
| Radiotherapy field |  |  |  |  |  |  |  |  |
| No PAO-radiotherapy  (incl. other infradiaphragmatic fields) | 26 | 6.3 | 4 | 8.7 | 9 | 6.3 | 13 | 5.8 |
| PAO | 162 | 39.1 | 1 | - | 18 | 12.6 | 143 | 63.6 |
| Dog Leg | 214 | 51.7 | 41 | 89.1 | 113 | 79.0 | 60 | 26.7 |
| Unknown field | 12 | 2.9 | - | - | 3 | 2.1 | 9 | 4.0 |
| Radiotherapy dose |  |  |  |  |  |  |  |  |
| No para-aortic radiotherapy | 26 | 6..3 | 4 | 8.7 | 9 | 6.3 | 13 | 5.7 |
| ≤26 Gy | 86 | 20.7 | 13 | 28.3 | 34 | 23.8 | 39 | 17.2 |
| 27-32 | 231 | 55.5 | 16 | 34.8 | 76 | 53.2 | 139 | 61.2 |
| >33 | 33 | 7.9 | 11 | 23.9 | 9 | 6.3 | 13 | 5.7 |
| Missing dose | 28 | 6.7 | 2 | 4.4 | 12 | 8.4 | 14 | 6.2 |
| Missing field, probably para-aortic | 12 | 2.9 | - | - | 3 | 2.1 | 9 | 4.0 |
| Median para-aortic dose, IQR | 26 | 26-26 | 30 | 25-40 | 26 | 25-26 | 26 | 26-26 |
| Median para-aortic dose, min/max | 26 | 17-60 | 30 | 16-60 | 26 | 21-46 | 26 | 17-51 |
| Diabetes in subcohort, % yes ‡ | 33 | 8.0 | 8 | 17.4 | 13 | 9.1 | 12 | 5.3 |

Distribution of total treatment (primary and follow-up) in DM subcohort for seminomas over treatment period.

‡ Number of DM cases with surgery only among seminomas in subcohort: *N*=1, 1976-1985.

| Supplemental tables S4. Treatment distribution by period in the DM subcohort. | | | | | | | | |
| --- | --- | --- | --- | --- | --- | --- | --- | --- |
| Panel B. All patients in subcohort for DM study. | | | | | | | | |
|  | **Overall**  ***N*=614** | | **1976-1985**  ***N*=93** | | **1986-1995**  ***N*=216** | | **1996-2007**  ***N*=305** | |
|  | ***N*** | **%** | ***N*** | **%** | ***N*** | **%** | ***N*** | **%** |
| Histology |  |  |  |  |  |  |  |  |
| Seminoma | 26 | 12.5 | 46 | 49.5 | 143 | 66.2 | 225 | 73.8 |
| Non-seminoma | 182 | 87.5 | 47 | 50.5 | 73 | 33.8 | 80 | 26.2 |
| Median age at diagnosis (SD) | 33.3 | 27.9-39.0 | 31.5 | 25.8-38.9 | 33.0 | 27.6-38.9 | 33.7 | 28.6-39.0 |
| Follow-up duration, years |  |  |  |  |  |  |  |  |
| Median | 14.6 | 9.6-21.3 | 28.7 | 20.0-31.6 | 20.4 | 17.6-24.0 | 10.9 | 8.0-13.5 |
| IQR |  |  |  |  |  |  |  |  |
| 0-4 | 43 | 7.0 | 7 | 7.5 | 7 | 3.2 | 29 | 9.5 |
| 5-9 | 126 | 20.5 | 3 | 3.2 | 19 | 8.8 | 104 | 34.1 |
| 10-14 | 149 | 24.3 | 6 | 6.5 | 10 | 4.6 | 133 | 43.6 |
| 15-19 | 107 | 17.4 | 7 | 7.5 | 61 | 28.2 | 39 | 12.8 |
| 20-24 | 101 | 16.5 | 17 | 18.3 | 84 | 38.9 | - | - |
| 25-29 | 52 | 8.5 | 17 | 18.3 | 35 | 16.2 | - | - |
| 30+ | 36 | 5.9 | 36 | 38.7 | - | - | - | - |
| Relapse, % yes | 30 | 4.9 | 7 | 7.5 | 6 | 2.8 | 17 | 5.5 |
| Radiotherapy field |  |  |  |  |  |  |  |  |
| No PAO-radiotherapy (incl. other infradiaphragmatic fields) | 208 | 33.9 | 35 | 37.6 | 82 | 38.0 | 91 | 29.8 |
| PAO | 164 | 26.7 | 1 | 1.1 | 18 | 8.3 | 145 | 47.5 |
| Dog Leg | 229 | 37.3 | 56 | 60.2 | 113 | 52.3 | 60 | 19.7 |
| Unknown field | 13 | 2.1 | 1 | 1.1 | 3 | 1.4 | 9 | 3.0 |
| Radiotherapy dose |  |  |  |  |  |  |  |  |
| No PAO- radiotherapy | 208 | 33.9 | 35 | 37.6 | 82 | 38.0 | 91 | 29.8 |
| ≤26 Gy | 86 | 14.0 | 13 | 14.0 | 34 | 15.7 | 39 | 12.8 |
| 27-32 | 233 | 38.0 | 16 | 17.2 | 76 | 35.2 | 141 | 45.2 |
| >33 | 48 | 7.8 | 26 | 28.0 | 9 | 4.2 | 13 | 4.3 |
| Missing dose | 28 | 4.2 | 2 | 2.2 | 12 | 5.6 | 12 | 3.9 |
| Missing field, probably PAO | 133 | 2.1 | 1 | 1.1 | 3 | 1.4 | 9 | 3.0 |
| Median para-aortic dose, IQR | 26 | 26-26 | 30 | 30-40 | 26 | 25-26 | 26 | 26-26 |
| Median para-aortic dose, min/max | 26 | 17-60 | 30 | 16-70 | 26 | 20-46 | 26 | 17-51 |
| Diabetes in subcohort, % yes‡ | 39 | 6.3 | 12 | 12.9 | 14 | 6.5 | 13 | 4.2 |

**Supplemental Figure S5. Radiotherapy dose distribution plots for seminoma and non-seminoma patients in DM subcohort.**

| Supplemental table S6. Standardized Incidence Ratios for DM compared to the general population for period 2002-2015. | | | | | | | | | | | | | | | |
| --- | --- | --- | --- | --- | --- | --- | --- | --- | --- | --- | --- | --- | --- | --- | --- |
|  | **Case-cohort (N=117)**  **Subcohort+ all cases**  **Period 2002-2015**  **Left censored** | | | | | **Case-cohort (N=117)**  **Subcohort+ all cases**  **Period 2002-2015**  **Left censored*** | | | | | **Cohort (N=117)**  **Period 2002-2015**  **Left censored*** | | | | |
|  | **O** | **SIR** | **95%CI** | **AER** | **95%CI** | **O** | **SIR** | **95%CI** | **AER** | **95%CI** | **O** | **SIR** | **95%CI** | **AER** | **95%CI** |
| Overall effect | 117 | 0.9 | 0.7-1.1 | -12.9 | -29.5-6.3 | 117 | 0.9 | 0.7-1.1 | -11.3 | -28-7.8 | 117 | 0.9 | 0.7-1.1 | 132.4 | -16.6-12.4 |
| Age at Dx |  |  |  |  |  |  |  |  |  |  |  |  |  |  |  |
| <35 | 46 | 0.9 | 0.7-1.2 | -3.7 | -12.7-7.4 | 46 | 0.9 | 0.7-1.2 | -2.9 | -11.8-8.2 | 46 | 0.8 | 0.6-1.2 | -5.1 | -13.7-5.7 |
| 35-39 | 27 | 0.8 | 0.6-1.2 | -9.0 | -28.1-13.6 | 27 | 0.9 | 0.6-1.2 | -8.6 | -25.7-13.9 | 27 | 0.8 | 0.5-1.2 | -12.6 | -30.6-11.4 |
| 40-50 | 44 | 0.9 | 0.6-1.2 | -11.0 | -35.1-19.3 | 44 | 0.9 | 0.7-1.2 | -9.9 | -34.0-20.3 | 44 | 1.0 | 0.7-1.3 | 0.7 | -25.0-32.9 |
| P-trend |  |  |  |  | 0.950 |  |  |  |  | 0.907 |  |  |  |  | 0.480 |
| P-heterogeneity |  |  |  |  | 0.970 |  |  |  |  | 0.953 |  |  |  |  | 0.618 |
| Period |  |  |  |  |  |  |  |  |  |  |  |  |  |  |  |
| 1976-1985 | 38 | 1.2 | 0.8-1.7 | 23.0 | -16.8-74.1 | 38 | 1.2 | 0.9-1.7 | 25.2 | -14.6-76.3 | 38 | 1.0 | 0.7-1.4 | 4.0 | -29.5-46.8 |
| 1986-1995 | 34 | 0.6 | 0.4-0.8 | -26.7 | -38.7--11.3 | 34 | 0.6 | 0.4-0.8 | -25.9 | -37.9- -10.5 | 34 | 0.7 | 0.5-1.0 | -17.9 | -31.8- -0.2 |
| 1996-2007 | 45 | 1.1 | 0.8-1.4 | 1.7 | -7.9-13.7 | 45 | 1.1 | 0.8-1.4 | 2.1 | -7.5-14.3 | 45 | 1.0 | 0.7-1.3 | -0.6 | -9.8-11.0 |
| P-trend |  |  |  |  | 0.746 |  |  |  |  | 0.727 |  |  |  |  | 0.892 |
| P-heterogeneity |  |  |  |  | 0.005 |  |  |  |  | 0.006 |  |  |  |  | 0.237 |
| Follow-up |  |  |  |  |  |  |  |  |  |  |  |  |  |  |  |
| 1-9 | 35 | 1.1 | 0.7-1.5 | 3.9 | -15.7-29.0 | 35 | 1.1 | 0.8-1.5 | 5.0 | -14.6-30.2 | 35 | 1.0 | 0.7-1.4 | 2.2 | -16.9-26.8 |
| 10-19 | 35 | 0.6 | 0.5-0.9 | -43.6 | -67.6—12.7 | 35 | 0.7 | 0.5-0.9 | -42.3 | -66.3—11.4 | 35 | 0.7 | 0.5-1.0 | -34.7 | -59.9- -2.4 |
| 20-30 | 36 | 1.0 | 0.7-1.4 | 4.3 | -54.6—12.7 | 36 | 1.0 | 0.7-1.4 | 8.2 | -50.3-83.0 | 36 | 1.0 | 0.7-1.4 | 1.9 | -57.2-77.0 |
| 30+ | 11 | 1.1 | 0.5-2.3 | 37.9 | -128.9-322.7 | 11 | 1.1 | 0.5-2.2 | 35.4 | -131.4-320.2 | 11 | 1.4 | 0.7-2.5 | 2.7 | -139.5- 245.6 |
| P-trend |  |  |  |  | 0.774 |  |  |  |  | 0.793 |  |  |  |  | 0.881 |
| P-heterogeneity |  |  |  |  | 0.114 |  |  |  |  | 0.109 |  |  |  |  | 0.341 |
| Attained age |  |  |  |  |  |  |  |  |  |  |  |  |  |  |  |
| 10-50 | 44 | 0.8 | 0.6-1.1 | -39.0 | -89.3-24.2 | 44 | 0.8 | 0.6-1.2 | -35.9 | -86.3-27.2 | 44 | 0.6 | 0.2-43.6 | -3.7 | -11.7-265.0 |
| 50-64 | 40 | 0.8 | 0.6-1.1 | -27.5 | -69.3-25.0 | 40 | 0.8 | 0.6-1.2 | -23.9 | -65-28.5 | 40 | 0.8 | 0.5-1.3 | -16.9 | -45.7-23.4 |
| 65+ | 35 | 1.0 | 0.7-1.5 | 32.4 | -207.8-345 | 35 | 1.1 | 0.7-1.5 | 36.3 | -203.8-349.0 | 35 | 0.6 | 0.3-6.2 | -55.3 | -105.4-685.9 |
| P-trend |  |  |  |  | 0.338 |  |  |  |  | 0.352 |  |  |  |  | 0.490 |
| P-heterogeneity |  |  |  |  | 0.551 |  |  |  |  | 0.582 |  |  |  |  | 0.520 |
| Radiotherapy |  |  |  |  |  |  |  |  |  |  |  |  |  |  |  |
| No | 19 | 0.6 | 0.4-1.0 | -15.7 | -26.5- -0.5 | 19 | 0.6 | 0.4-1.0 | -15.2 | -26.1- -0.4 | 19 | 0.4 | 0.3-0.6 | -29.6 | -37.3- -18.7 |
| Yes | 98 | 1.0 | 0.8-1.2 | -2.6 | -13.3-9.9 | 98 | 1.0 | 0.8-1.2 | -1.6 | -12.4-10.8 | 98 | 1.1 | 0.9-1.4 | 8.5 | -4.1-23.1 |
| P-heterogeneity |  |  |  |  | 0.102 |  |  |  |  | 0.097 |  |  |  |  | <0.001 |

*** A smoothed regression model based on gender and age was used to model the time-dependent expected diabetes incidence in the general population.**

| Supplemental table S7. Risk of DM by treatment: complete case analyses.^ǂ^ | | | | | |
| --- | --- | --- | --- | --- | --- |
|  | **Unadjusted** | | | **Adjusted for age** | |
|  | ***N* cases** | **HR** | **95%CI** | **HR** | **95%CI** |
| Radiotherapy field |  |  |  |  |  |
| No PAO-radiotherapy | 25 | **1** | **ref** | **1** | **ref** |
| PAO-radiotherapy‡ | 115 | 2.4 | 1.5-3.8 | 1.9 | 1.2-3.2 |
| Radiotherapy dose** |  |  |  |  |  |
| No para-aortic radiotherapy | 23 | **1** | **ref** | **1** | **ref** |
| ≤26 Gy | 77 | 2.9 | 1.6-5.3 | 2.2 | 1.2-4.2 |
| 27-32 | 27 | 2.6 | 1.6-4.3 | 1.8 | 1.0-3.1 |
| ≥33 | 13 | 1.6 | 0.8-3.3 | 1.2 | 0.5-2.7 |
| Smoking status at TC diagnosis |  |  |  |  |  |
| No | 81 | **1** | **ref** | **1** | **ref** |
| Yes | 59 | 1.2 | 0.7-1.8 | 1.2 | 0.8-1.9 |
| BMI at TC diagnosis§ |  |  |  |  |  |
| < 30 kg/m^2^ | 117 | 1 | ref | 1 | ref |
| 30+ kg/m^2^ | 23 | 2.4 | 1.2-4.6 | 2.3 | 1.1-4.8 |

ǂ In the complete case analysis, 140 DM cases (87.0%) are included with complete data on TC treatment. For 569/616 subcohort patients (92.3%), complete information on primary and follow-up radiotherapy field and dose was available.

‡ Three patients who had abdominal radiotherapy other than para-aortic or dog-leg radiotherapywere excluded from the complete case analysis.

** P=heterogeneity:0.06
